# Empowering pharmacoinformatics by linked life science data

**DOI:** 10.1007/s10822-016-9990-4

**Published:** 2016-11-09

**Authors:** Daria Goldmann, Barbara Zdrazil, Daniela Digles, Gerhard F. Ecker

**Affiliations:** Department of Pharmaceutical Chemistry, University of Vienna, Althanstraße 14, 1090 Vienna, Austria

**Keywords:** Data extraction, Data curation, Data integration, QSAR, Computer-aided drug discovery, TRPV1, Pharmacophore modeling

## Abstract

With the public availability of large data sources such as ChEMBLdb and the Open PHACTS Discovery Platform, retrieval of data sets for certain protein targets of interest with consistent assay conditions is no longer a time consuming process. Especially the use of workflow engines such as KNIME or Pipeline Pilot allows complex queries and enables to simultaneously search for several targets. Data can then directly be used as input to various ligand- and structure-based studies. In this contribution, using in-house projects on P-gp inhibition, transporter selectivity, and TRPV1 modulation we outline how the incorporation of linked life science data in the daily execution of projects allowed to expand our approaches from conventional Hansch analysis to complex, integrated multilayer models.

**Electronic supplementary material:**

The online version of this article (doi:10.1007/s10822-016-9990-4) contains supplementary material, which is available to authorized users.

## Introduction

The methodology of 3D quantitative structure–activity relationships (3D-QSAR) established in 1988 by Richard Cramer could be referred to as one of the first Computer-Aided Drug Design (CADD) strategies to facilitate drug discovery [1]. Since then, the introduction of pharmacophore modeling, molecular docking, molecular dynamics simulations, and free energy calculations helped to identify and to analyze the molecular basis of protein–ligand interactions. Recent progress in experimental methods, such as X-ray crystallography, NMR spectroscopy, and Cryo electron microscopy, increases the number and quality of the protein structures available, and thus fosters structure-based design strategies. Lately, the Worldwide PDB (wwPDB) organization hosts multiple depositories for 3D protein and nucleic acid structures: the Protein Data Bank (http://www.rcsb.org/; RCSB PDB [2]), the Protein Data Bank in Europe (PDBe), Protein Data Bank Japan (PDBj), the Biological Magnetic Resonance Data Bank (http://www.bmrb.wisc.edu/; BMRB [3]), and the Electron Microscopy Data Bank (http://www.emdatabank.org; EMDataBank [4]). In addition to those, small-molecule organic and metal–organic crystal structures are deposited in the Cambridge Structural Database (http://www.ccdc.cam.ac.uk/; CSD [5, 6]) to be exploited by the scientific community.

With respect to small molecule pharmacological (and related) data, numerous open data sources and platforms are now facilitating their access, integration and re-use. Pre-competitive alliances aid in the development of proper ontologies, and the mapping to existing ontologies and vocabulary, which are needed for seamless data integration. Such initiatives alleviate data access by removing some of the main hurdles in the Life Sciences domain: data heterogeneity in all its aspects (even concerning legality and licensing issues). By providing the data via a stable infrastructure, the Open PHACTS Discovery Platform (https://www.openphacts.org/open-phacts-discovery-platform) facilitates the processing of even more complex research questions, going beyond the concepts “target-ligand-bioactivity”. Postprocessing by a modular pipelining tool (e.g. KNIME [7] https://www.knime.org or Pipeline Pilot [8] http://accelrys.com/products/pipeline-pilot/) complements the workflow of data curation and analysis. Using open source software tools such as WEKA [9], RDKit (http://www.rdkit.org), and SCIKIT [10], predictive models can be built conveniently right inside the data curation workflows or as a separate instance.

**Fig. 1:**
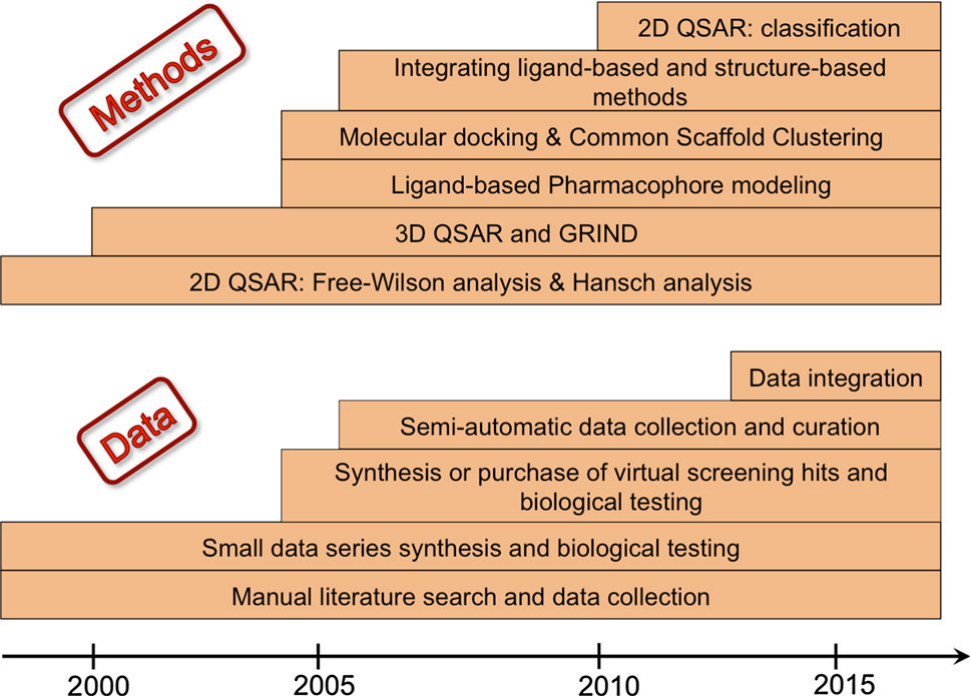
Evolution of the applied computer-aided drug design methods and data access practices in the Pharmacoinformatics Research group of Vienna

Looking 10 years back, academic research groups focused their research on small data sets of compounds originating from collaborators within their closest research environment: either within the academic institution, a close academic collaborator, or a single pharma company (in the latter case without disclosing the compounds’ chemical structures). Computational chemists primarily applied 2D or 3D QSAR analysis to propose chemical modifications to guide synthesis. Currently the pharmacological, biological, and structural data are shared internationally within large EU-projects encouraging a dialog between specialists of different fields. Additionally, public–private initiatives facilitate close collaborations between academia and industry, giving access to proprietary data in order to develop and test novel computational methods on them. Thus, academic CADD is increasingly looking into topics which benefit from the unprecedented access to data, such as phenotypic annotations, in silico tools to facilitate drug repurposing, translational and precision medicine and provides computational solutions there, such as tailored workflows [11–13]. In this contribution we seek to demonstrate how all these developments influenced our (the Pharmacoinformatics Research Group at the Department of Pharmaceutical Chemistry, University of Vienna) way of approaching challenging research questions (Fig. 1).

## From synthesis of small *in*-*house* libraries to first computational approaches

**Fig. 2:**
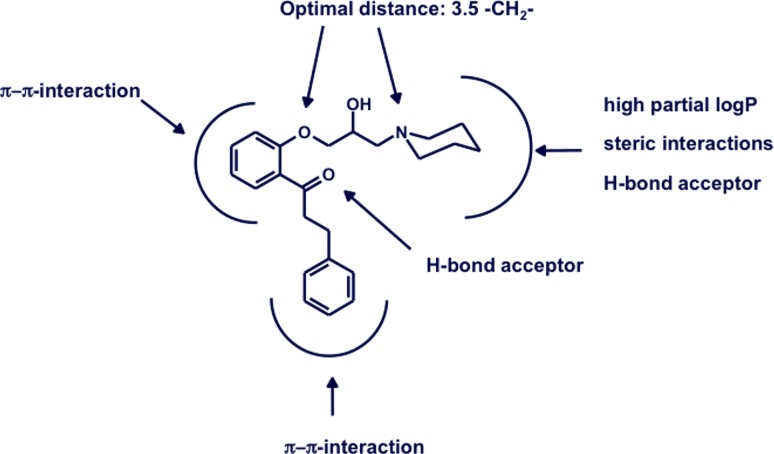
Summary of the results of structure–activity relationship studies on propafenon-type inhibitors of P-gp Adapted from [64]

With the ground breaking contributions of Hansch, Fujita, and Seydel, correlations of differences in chemical structures with differences in respective biological activities for small, congeneric compound series became a widely used tool for lead optimisation. As this classical quantitative structure–activity relationship (QSAR)—often referred to as Hansch analysis—not necessarily requires expensive hard- and software, it was free to everyone. Ideally, based on hypotheses such as “the para-substituent on this aromatic ring influences biological activity mainly via electron donating properties”, small, focused sets of compounds were synthesized, tested, and the correlation between electron donating property and pIC_50_ values were seen as proof of hypothesis. Numerous medicinal chemistry groups started to include these approaches into their daily routine in order to rationalise their compound design. Also our work on propafenone-type inhibitors of the drug efflux pump P-glycoprotein (P-gp) started with very simple Hansch analyses, mainly based on logP as descriptor [14]. Expanding this to descriptors for e.g. hydrogen bond (H-bond) acceptor strength enabled to derive a hypothesis whether or not this type of compounds interact in charged or uncharged form [15]. Including size and distance finally allowed us to draw a picture as provided in Fig. 2, which summarizes the result of synthesizing and testing of more than 200 propafenone analogs [16]. This not only led to P-gp inhibitors which show three orders of magnitude higher activity than the parent compound propafenone (IC_50_ propafenone = 3 µM, IC_50_ GPV0576 = 5 nM), it also laid the ground for application of more complex CADD methods. These comprise, among others, the development of CoMFA and Hologram QSAR models [17], the development of pharmacophore models [18], as well as the application of self organising maps [19]. Both the pharmacophore model and the self organising map were further used for virtual screening, thereby identifying new, structurally unrelated P-gp inhibitors. This already marked a further step in the development of the group, the proof of hypothesis by prospective in silico screening followed by experimental testing, rather than by retrospective correlation analysis.

With the first X-ray structures of ABC-transporters published, also structure-based design strategies were pursued. However, it should be noted that we still lack the structure of human P-gp, and that basically all structure-based studies are conducted with homology models based on the mouse P-gp structure [20] and its refined version [21]. Also in this case the propafenone data set served as valuable basis for docking experiments. As transporters generally show high plasticity, and in case of ABC-transporters the ligand binding area is huge (estimates go up to 8000 Å^3^), conventional protocols, which rely on scoring functions for detecting the “right” binding mode, are likely to fail. Thus, we established what we call “experimental data guided docking”. Briefly, a small set of compounds which show a distinct SAR pattern are docked, and the poses retrieved are clustered according to the common scaffold of the compound series. Subsequently, the clusters obtained are analysed with respect to their ability to explain the SAR pattern. This approach has been successfully applied for propafenones and P-gp [22], tricyclic antidepressants and the serotonin transporter (SERT) [23], benzodiazepines and the γ-aminobutyric acid receptor type A (GABAA) [24], and tiagabine analogs and γ-aminobutyric acid transporter type 1 (GAT1) [25]. Experimental support for the docking poses is retrieved by transforming the pose to a structure-based pharmacophore model using LigandScout [26], followed by in silico screening of a vendor library and buying and testing the top ranked hits. This workflow is now routinely applied in all structure-based studies conducted in our group (see also TRPV1 use case below). It exemplifies how simple Hansch analyses progressed towards a complex workflow integrating ligand- and structure-based design methods in order to target transmembrane transport proteins.

## From manual extraction and curation of literature data to (semi-) automatic data retrieval, curation, and processing within the framework of pre-competitive alliances

Since more than a decade, the biomedical and healthcare sectors are experiencing the consequences of the digital revolution. As more and more data becomes openly available, we are facing challenges regarding data management, harmonization (standardization), integration, and storage. Guidelines and initiatives trying to overcome some of these challenges are under active development by many consortia across Europe (e.g. Open PHACTS [27]) and US (e.g. OBO foundry [28]). However, from the perspective of an individual data scientist, the daily challenge lies in finding the most suitable data retrieval, curation and processing strategy in order to answer the underlying research question(s) and to draw meaningful conclusions from it. These decisions are influenced by many factors, such as the nature and size of the primary data to be retrieved, the number of layers and complexity of information/data to be integrated, the level of detail to be achieved, as well as the common scientific culture within the lab or research entity.

Being embedded within an academic research environment, we have to deal with the fact that pre-clinical research data coming from the open domain is generally less well structured and curated than clinical data [29]. Thus, for the purpose of generating predictive models, it will in many cases appear inevitable to manually extract the pharmacological and other biomedical data directly from its primary source, i.e. from its scholarly publication. In that way, the detailed descriptions of underlying bioassays as well as recommendations regarding the cutoffs for separation of actives and inactives by the original authors of the data sets can be collected.

In the (recent) past, we have applied this *modus operandi* on various use cases, however with minor differences regarding the exact data extraction and curation protocols. Klepsch et al. [32] used two publications [30, 31] in order to collate their data set for P-gp inhibitor classification models. However, for one of these papers [30], sixty primary data sources originally served the authors for its compilation. Thus, we could make use of the curation efforts of other scientists with respect to data gathering as well as considerations regarding a reasonable cutoff (needed for active/inactive class assignment). Although different bioassay protocols were used for measuring the compounds’ pharmacological activities, by incorporating multiple individual criteria for class assignment (the IC_50_, percent inhibition values, and the multidrug-resistance ratio), the data points could be joined to one big data set composed of approx. 1600 unique molecules.

While the inter- and intra-comparability of bioassays is a well known challenge in data-driven science, still there is no general agreement or guideline on how to accurately compile a data set from pharmacological data that has been produced under different assay conditions. For smaller data sets, an isolated target or a target family, one might take the effort to manually map bioassays according to their protocols (narrative descriptions) in databases (e.g. ChEMBLdb) or in the primary literature. Earlier, we have reported our annotation effort for human P-gp inhibitors [33] and proposed strategies to make use of existing ontologies for mapping identical and combinable assays [34] via nanopublications [35, 36].

Going a step further, a data compilation exercise for breast cancer resistance protein (BCRP) inhibitors [37] led us to generate tailored cutoffs. These cutoffs were adjusted to the specific sources and bioassays by performing an in-depth analysis of pharmacological bioactivities for a set of reference compounds across different papers and assays. In addition to peer-reviewed articles, high-throughput screening (HTS) data from Pubchem was included making use of inherent labels (“Active”, “Inactive”, “Inconclusive”, “Unspecified”) for assigning the classes.

However, such extensive curation efforts might often appear unfeasible for large data sets. There we sometimes have to deal with a certain degree of uncertainty of bioactivity data if we want to fully exploit all available data. Kalliokoski et al. [38] have shown that the error introduced by mixing IC_50_ data from different assays only adds a moderate amount of noise when comparing different measurements of the same compound-target pairs. Moreover, such intravariabilities (same compound target pair, same bioactivity endpoint, different assays) seem to be in the same range as intervariabilities (same compound target pair, different bioactivity endpoints, different assays) as we have successfully demonstrated for IC_50_ and K_i_ measurements of human serotonin and dopamine transporter ligands [11]. Thus, even when mixing IC_50_ with K_i_ data, the error introduced might be tolerable, depending on the final granularity that shall be achieved and the usage of the dataset (i.e. for retrieving selectivity tendencies).

Regarding data cleaning and curation, protocols differ between research groups and also between different projects. The choice of software tools to use for these cleaning and standardization steps is very much dependent on their availability. Open source tools, such as the Chemical Validation and Standardization Platform (CVSP) [39], developed by the Royal Society of Chemistry (RSC), will hopefully facilitate validation and standardization of chemical structure data sets from various sources. Moreover, providing a platform for performing all curation steps within the same environment might diminish mistakes occurring by incompatibilities between data formats of different platforms.

While it might be worth the effort to manually curate a data set for one or two selected targets of interest, there is a need for a (semi-) automatic procedure for the simultaneous extraction and curation of many targets or for investigation of relationships between data of those targets. By managing and scientifically participating in the Open PHACTS project [27] from 2011 until 2016, and being a partner in its successor organization the Open PHACTS Foundation (http://www.openphactsfoundation.org/), our group got increasingly engaged in big data management, integration, curation, and mining. Retrospectively, these activities influenced tremendously the way we are performing data compilation, but also the kind of scientific questions we are targeting. Since one of the very first activities of the project was the collection of exemplary scientific research questions [40], commonly tackled within the research entities both in academia and in pharma companies, we got an immediate feeling of the actual requirements driving a collaborative research environment. By making use of the expertise of a large multidisciplinary consortium, we could harness research strategies from both worlds (academia vs. pharma, or IT specialist vs. application scientist). In addition, such pre-competitive alliances are providing a framework that facilitates data sharing in a way that the extent of shared data and especially its metadata (data about data; the underlying description of the data) will be larger. Thus, any spurious findings (like data inconsistencies, outliers, etc.) would also be detected earlier, [29] which has an impact on the quality of the results.

With the emergence of specialized Open PHACTS Pipeline Pilot components and KNIME nodes (https://dev.openphacts.org/resources) it became feasible to simultaneously query for multiple targets or even pathways of interest within a single data pipelining environment, which as well provides nodes for data curation and analysis. We successfully demonstrated the usability of the Open PHACTS Discovery Platform in conjunction with pipelining tools for solving drug discovery relevant research questions by performing data compilation for whole regulatory pathways of interest [41].

Applying these methods, we integrated open data with manually retrieved literature data [37], and were able to determine compound overlap for human P-gp and BCRP inhibitors within a semiautomatic data curation workflow. This allowed us to generate data sets for studying their selectivity profiles [42]. Subsequently, the data sets of P-gp-selective inhibitors, BCRP-selective inhibitors, and P-gp/BCRP dual inhibitors served to establish different multi-label classification models, which in turn revealed important molecular features driving selective or polyspecific inhibitory activity. Elaborating this idea further, a workflow of this kind could be used for building multi-label data sets of any set of pharmacological targets for which there is data available either in the open domain or in-house (even for a whole protein family).

With the idea that certain molecular features might trigger selectivity, two other phylogenetically related transporters of interest to our group, the human serotonin (hSERT) and dopamine transporters (hDAT), served for a combined data mining/in silico modeling study [11]. Data was extracted solely from the public domain for this case study, but cutoffs for the separation of actives and inactives were tailored according to targets and bioactivity endpoints (K_i_ and IC_50_). The aim was to investigate the ability to retrieve consistent SAR series by the clustering of compounds according to their common scaffolds in an automatic fashion. These compound series further served for SAR and modeling studies. In addition, we were interested in the potential of such clustering methods to extract scaffold series with a pronounced selectivity trend towards hSERT/hDAT. Concludingly, automatic scaffold clustering might serve as useful tool to get first hints on privileged scaffolds. Still, as our study showed, it is always advisable to combine scaffold clustering methods with similarity searches (such as a common substructure search).

In summary, the combined use of open and literature data with workflow tools most probably represents the best option to generate data sets of suitable size and quality to be analyzed further by machine learning and other in silico modeling methods. It will be necessary to elaborate some standard operating procedures within the data mining community, describing best practice for data curation (such as the mapping of bioactivities for different bioassay and accurate cutoff setting).

## From large data extraction to the pharmacological action on the molecular level—a recent use case

Combine, curate, exploit, abstract, test, project, and validate are the seven pillars to guide a computer-aided drug design project in our group. In the paragraphs below, we are using TRPV1 as an example to demonstrate common pitfalls that we face, as well as the methods and tools we are using for (1) collecting relevant pharmacological data, (2) filtering them from artifacts, (3) building computational models for prediction of novel compounds, (4) selecting the best model, (5) prioritizing virtual hits for experimental testing, (6) using experimental ligand-based data to guide molecular docking studies, and (7) identifying plausible binding modes of novel therapeutically relevant compounds.

The molecular target of interest was the Transient Receptor Potential Vanilloid Type 1 (TRPV1)—an ion channel that senses potentially harmful chemical, mechanical, and thermal stimuli in the peripheral nervous system (PNS) and causes pain perception. Therefore, its antagonists could provide an alternative for the treatment of chronic and neuropathic pain as non-opioid analgesics [43, 44]. Since the structure of the TRPV1 channel was not yet resolved when the project started, we relied on numerous mutagenicity reports [45] and a few homology modeling studies [46, 47] to locate a putative binding site. Additionally, extensive pharmacological reports from PubMed [48, 49] provided insights into the structure–activity relationships (SAR) of diverse subsets of agonists and antagonists of the receptor.

To collect a data set of TRPV1 ligands published until 2011 we used ChEMBLdb v.13 [50] and extracted pharmacological data for over 2300 chemicals. Since ChEMBLdb excerpts chemical structures and bioassay descriptions directly from the underlying publication, assay descriptions are heterogeneous and we therefore carefully filtered the data from artifacts [51]. We manually compared those descriptions provided in the underlying publications and prioritized 408 antagonists (reference data set) measured as competitive inhibitors of the capsaicin activation of the human TRPV1 receptor expressed in HEK293 cell lines (A detailed description of the filtering protocol is available in [51]). This way we ensured that compounds in the data set are occupying the same binding site as the reference agonist, capsaicin, and could be used in future structure-based studies. At present, an automatic extraction of pharmacological data using ChEMBLdb REST nodes in KNIME returns over 4700 ligands of human TRPV1, from which 2019 ligands have reported IC_50_ values. Stepwise automatic curation (following the protocol from 2011) yields 403 unique compound structures, a slightly lower number than we extracted by manual comparison of assay protocols. These data contain 240 novel compounds, published between 2011 and 2015. However, 244 compounds, which we included in our reference data set in 2011 by manual comparison of assay protocols, were missed since their assay descriptions in ChEMBLdb are scarce, i.e. they lack reference terms like ‘capsaicin’ or ‘HEK293’. The KNIME workflow used for extraction and curation of pharmacological data for TRPV1 is provided under http://www.myexperiment.org/workflows/4915.html. Although the databases encourage the users to deposit their data using defined ontologies, this use case confirms that automatic data extraction and curation is imperfect due to existing scarce data descriptions. However, the protocol for the automatic data extraction is much more efficient and provides access to high quality, though limited, data within about one hour depending on the speed of your internet connection.

While building computational models on the data sets collected from open data sources, one has to pay attention to the following aspects: chemical diversity, data completion, size of the data set, distribution of the activity values, and the threshold for defining activity classes. The TRPV1 antagonists in our data set originated from structure–activity relationship (SAR) studies and represented several populated islands in the ocean of the possible chemical space [52]. The bioactivity values (IC_50_) in the data set ranged from low nM to high μM, and we chose a threshold of 100 nM to separate actives from inactives based on the IC_50_ value of the reference antagonist capsazepine, which was determined in the same experimental protocol.

The growing popularity of machine learning algorithms in the chemoinformatics community motivated us to build classification models on our TRPV1 data set along with other in-house data sets [32, 37, 53, 54]. While probing our data set with several machine learning algorithms and descriptors, we noticed that the models were not ideal for virtual screening, i.e. the search for novel compounds (accuracy between 60 and 70%). Furthermore, we detected several compounds that were constantly misclassified. They originated from the same chemical series and possessed only small differences in the substitution patterns of their scaffolds and hence showed similar descriptor values. Therefore, these minor differences could not be distinguished by classification algorithms. However, 3D GRID independent QSAR (GRIND) modeling [55] allowed to capture the properties of the specific substituents according to their influence on activity and to build satisfactory models for distinct chemical compound series.

The breakthrough for the TRPV1 use case was achieved with the application of ligand-based pharmacophore clustering, which allowed to abstract common features of the antagonists into several ensembles of pharmacophore models [56]. To select the models for virtual screening, we performed thorough computational validation with data sets of inactive TRPV1 antagonists, customized decoy sets, and active compounds in clinical trials. Virtual screening of a vendor database through the five best models led to a hit list of 1909 compounds selected from more than 300000. Since the vendor database consisted of numerous compound series, in which individual series share the same core and vary in the substituents, we hypothesized, that if the pharmacophore of the core matches our pharmacophore models, then at least a fraction of compounds sharing this core should appear in the hit list. The exception would be when the substituents of the core scaffold are bulky and clash with the exclusion volume spheres, which represent borders of a hypothetical binding pocket in a pharmacophore model. However, the cores of several compounds occurred only once in the hit list, while having numerous representatives in the vendor database. Those compounds alerted us by being singletons in the hit list while having non-bulky neighbors in the database and we considered them as potential outliers. Finally, we picked 12 top-scored compounds from the series that were enriched in the hit list and performed in vitro testing for them. Two of the compounds showed antagonistic activity against capsaicin activation of the TRPV1 channel and thus allowed us to confirm two of five pharmacophore models.

We are convinced, that experimental testing should serve as a proof of concept and ultimate validation of the selected computational approach. Currently, each of our computational projects, be it on ion channels, such as TRPV1 [56] and GABAA [24], or transporters, such as P-gp [unpublished], BSEP [37], BCRP [57], and GAT3 [unpublished], is validated in vitro either in-house or outsourced.

Coming back to the TRPV1 use case, we were intrigued to explore, whether we could use the two experimentally confirmed ligand-based pharmacophore models to guide validation of molecular docking and, ultimately, identify possible binding modes of the TRPV1 antagonists. In this way we used molecular docking as a structure-based in silico method for identification of important protein–ligand interactions. Exact parameters and specific settings used in the study are provided in the Electronic Supplementary Material (ESM). For our computational experiments highly active representatives of those two pharmacophoric clusters were used, which led to the experimentally confirmed models (Table ESM1). These compounds incorporated either isoquinoline or quinazoline substructure as a common scaffold and we further refer to them as class 1 (isoquinolines) and class 2 (quinazolines) TRPV1 antagonists, respectively (Figure ESM2 and ESM3). To be able to distinguish important protein–ligand interactions, we also performed molecular docking of the lower active compounds sharing the same common substructures, which did not fit our ligand-based pharmacophore models.

Since a high resolution structure of the TRPV1 bound with its ligands was not available at the time (structures of the TRPV1 bound with capsaicin, RTX and capsazepine were determined in June 2016), we used a recently released structure of the rat ortholog of TRPV1 in the apo state as a template for the homology modeling of the human TRPV1 [58] (Figures ESM4–ESM7). Competitive antagonists replace agonists in their binding pocket, which allows strict definition of the binding site in the transmembrane region on the interface of two adjacent subunits, which is also supported by numerous mutational studies [45] (Figure ESM8). Furthermore, those antagonists stabilize the receptor in the closed state, and thus information from the ligand-receptor complexes would provide a binding hypothesis for high affinity antagonists.

For identification of probable binding modes we used the experimental data guided docking approach outlined above. Analysis of the protein–ligand interaction fingerprints suggested, that the most active TRPV1 antagonists form a H-bond with the hydroxyl of the Thr550 and show van der Waals interactions with the residues Glu570 and Ile573 (Figure ESM9). Other residues of the binding pocket showed different patterns in the interaction with the two classes of antagonists, which suggests that isoquinolines and quinazolines adopt different binding modes while bound to TRPV1.

**Fig. 3:**
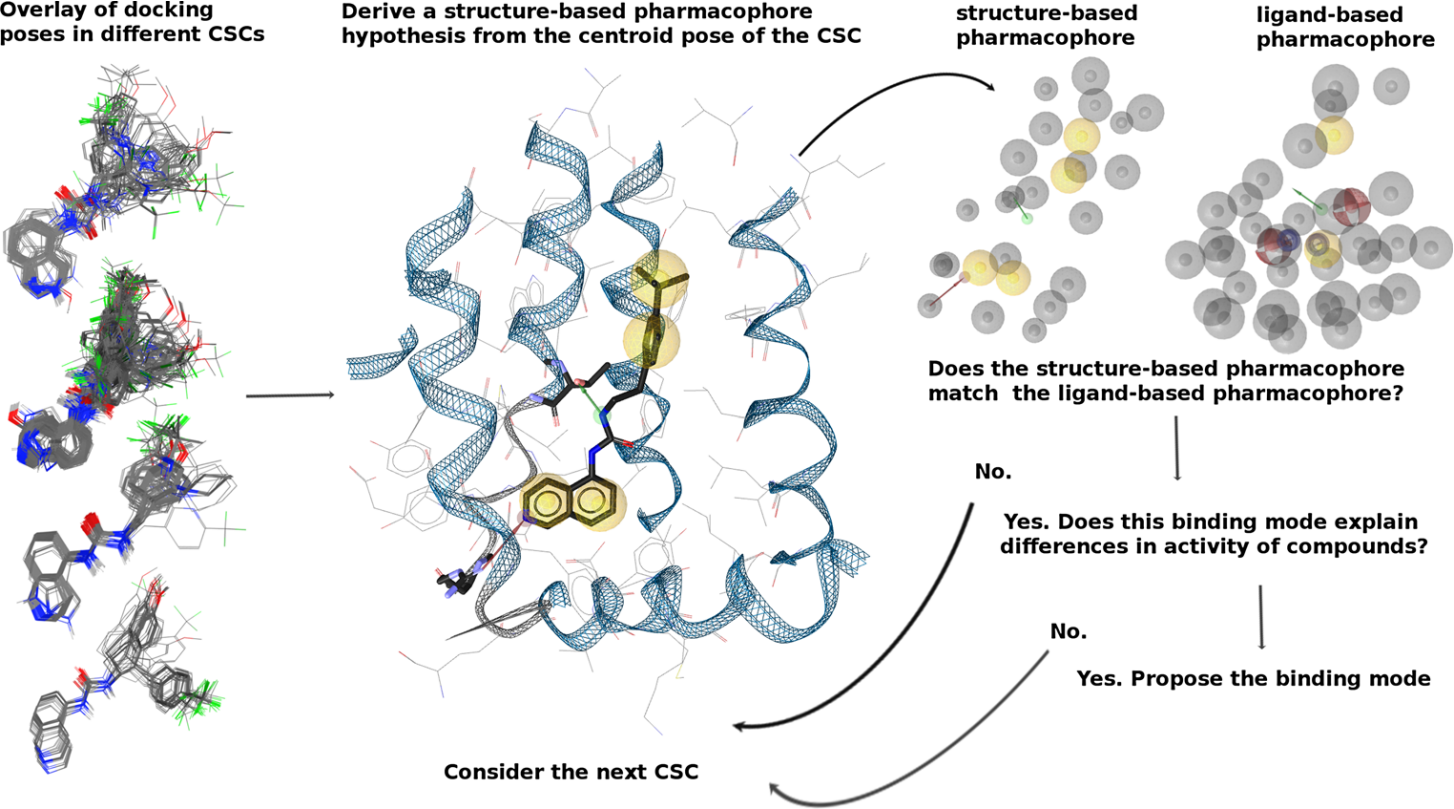
Protocol for evaluation of docking poses with structure-based and ligand-based pharmacophore models; CSC stands for common scaffold cluster

Subsequently, we applied the common scaffold clustering protocol for each set of ligands separately. According to the hypothesis of a common binding mode, structurally related compounds with high affinity should display similar orientation in the binding pocket and allow identical ligand-receptor interactions. Therefore, it is only worth to consider those clusters that include the majority of the poses of higher active compounds (Figure ESM10 and ESM11). In case of TRPV1, in addition to our standard protocol, for each common scaffold cluster we compared a structure-based pharmacophore generated from its centroid pose with the validated ligand-based pharmacophore models described previously (Fig. 3). This adds an additional layer in computational validation of the binding modes and allows to check whether the binding modes, i.e. structure-based pharmacophores, allowed discriminating between ligands with higher and lower activity.

**Fig. 4:**
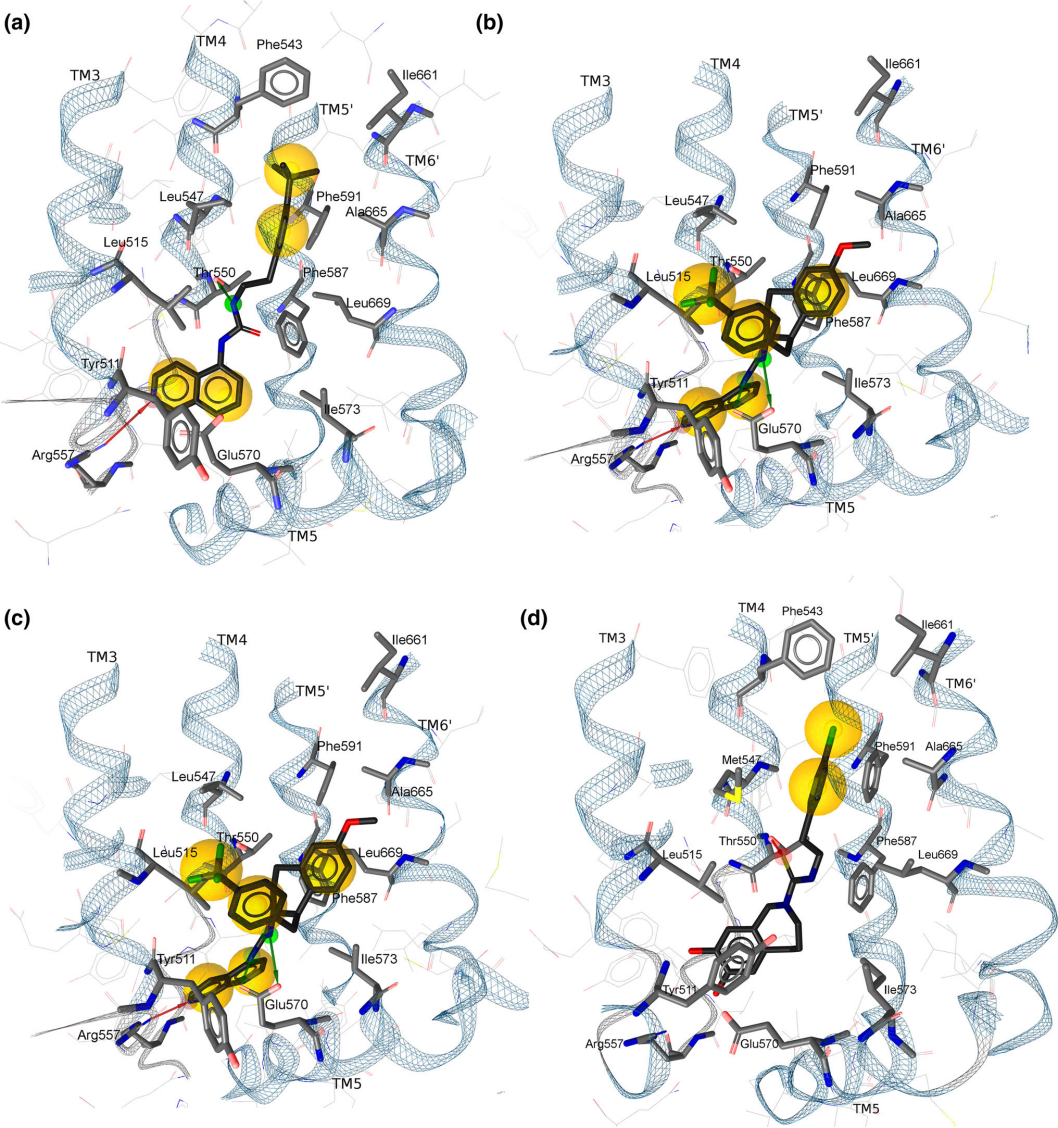
Binding mode hypothesis for TRPV1 antagonists; spheres represent hydrophobic interaction and arrows denote H-bonds (**a**) the first proposed binding mode of isoquinolines (**b**) the second proposed (but later rejected) binding mode of isoquinolines (**c**) proposed binding mode of quinazolines (**d**) binding mode of capsazepine in the structure with PDB ID 5IS0

For the TRPV1 antagonists of class 1 we predicted two possible orientations of the ligands in the binding pocket, i.e. two possible binding modes. In both cases the isoquinoline moiety was located in the lower part of the pocket. In the first mode, the molecule of the ligand was stretched in the binding pocket along trans-membrane helix 4 (TM4) of one of the receptor subunits and its urea linker was forming a H-bond with Thr550; the hydrophobic tail of the molecule was pointing towards the upper hydrophobic pocket (Fig. 4a). In the second mode, the linker and the hydrophobic tail of the ligand were pointing towards the subunit–subunit interface, thus forming a H-bond with Glu570 and van der Waals interaction with the residues of the adjacent subunit (Fig. 4b). In addition, we saw that the isoquinoline moiety was forming a H-bond with Arg557.

For the class 2 TRPV1 antagonists we observed that highly active antagonists were also stretched along the TM4 showing strong van der Waals contacts with the residues in the upper lower parts of the pocket. Additionally, quinazolines showed the same H-bond interaction with Thr550, which we observed for isoquinolines. Finally, bulky substituents of several quinazoline antagonist formed strong hydrophobic interactions with the residues of the adjacent subunit (Fig. 4c).

Summarizing, for both classes of TRPV1 antagonists we observed a H-bond with Thr550 and strong van der Waals interactions with hydrophobic residues in the lower and upper parts of the pocket. Our predictions are supported by two consecutive publications from independent groups in the last year [59, 60]. Yang et al. showed that interaction with Thr550 is essential for binding of an agonist capsaicin, however, a H-bond with Glu570 translates into gating of the channel. This finding allowed us to confirm our hypothesis of a common binding mode and to discard one of the binding mode hypotheses of the isoquinolines. Very recently, in May 2016, Gao et al. [60]. determined several structures of the rat ortholog of TRPV1 bound with an agonist, capsaicin, and an antagonist, capsazepine, and released them to the community. Capsazepine, when bound to the TRPV1, is stretched along TM4 and forms a H-bond with Thr550, reaffirming our hypothesis of the binding mode of competitive TRPV1 antagonists (Fig. 4d).

This use case convincingly demonstrates that multilayer, integrated approaches allow challenging targets such as transmembrane ion channels or transporters to be investigated via in silico modeling. This methodology of enriching structure-based analysis with ligand-based modeling, whereby the latter may be based on carefully curated public data, is now considered as standard operating procedure in our group when approaching new targets, such as human L-type amino acid transporter (LAT1).

## Outlook

The participation of our group in projects funded by the Innovative Medicines Initative, such as Open PHACTS, eTOX, and K4DD, increased the awareness of topics relevant to the pharmaceutical industry. Our focus thus gradually shifted from QSAR studies on small data sets and one target to integrated models applying several conceptionally different approaches to derive validated hypotheses for in silico screening, ending up at multilayer in silico models for prediction of in vivo toxicity [54]. This requires tools to automatically create high quality data sets out of the large amount of data available. However, making use of data sets available on the world wide web also introduces new challenges. For data users, it is important to give credit to the original data providers. Especially if different data sources are linked, it is important to make sure to always preserve its full provenance. For the data provider, it is important to specify what can be done with the data by providing an appropriate license. Basic guidelines which should aid the re-use of scholarly data were recently introduced as the FAIR data principles (Findability, Accessibility, Interoperability and Reusability) [61]. Nevertheless, licenses are not only important for the data, but also for the software used. While previously only the results of computational models were shared (together with a description of the methods), the tendency to share workflows to perform the calculations sets a new focus on open software. For example, a model created with descriptors calculated by proprietary software will only be of use for those who have the software license as well.

The availability of an increasing number of different life science data sets, and methods to link the entities in the data as well as new methods to mine the data, poses new challenges, but also offers many new opportunities to solve complex research questions, such as personalized medicine. A typical example might be the Open PHACTS project. A basic set of typical questions prioritized by the consortium members was the basis for the initial life science data sets integrated in the Open PHACTS Discovery Platform, focusing heavily on bioactivity data for protein targets [40]. With the availability of phenotypic screening data [62], this focus changed to include more complex biological read-outs. Here, linked life science data can help to interpret the outcomes of phenotypic screening runs and annotate possible targets [12]. Very recently, the platform was also used by our group for a repurposing approach [63], demonstrating the almost unlimited possibilities of linked life science data combined with workflow tools.

## Electronic supplementary material

Below is the link to the electronic supplementary material.

Supplementary material 1 (PDF 1994 kb)
