# Supplementary material for: Empowering pharmacoinformatics by linked life science data
Source: J Comput Aided Mol Des. 2016 Nov 9;31(3):319–28. doi: 10.1007/s10822-016-9990-4 (PMC5385323; doi:10.1007/s10822-016-9990-4)
Supplement: Supplementary file 1 — Supplementary material 1 (PDF 1994 kb) [file 10822_2016_9990_MOESM1_ESM.pdf]

Supplementary Material

Empowering Pharmacoinformatics by linked life science data

*Daria Goldmann§, Barbara Zdrazil§, Daniela Digles, and Gerhard F. Ecker**

University of Vienna, Department of Pharmaceutical Chemistry, Division of Drug Design and Medicinal Chemistry, Pharmacoinformatics Research Group, Althanstraße 14, A-1090 Vienna, Austria.
**Corresponding author:*
e-mail: gerhard.f.ecker@univie.ac.at; phone: +43 1 4277 55110; fax: +43 1 4277 855110
§ these authors contributed equally to this work

Computational Methods

Ligand preparation

Chemical structures of molecules 1 to 18 (Table ESM, Figure ESM2 and Figure ESM3) were drawn using MarvinSketch v6.3.0 of ChemAxon (ChemAxon, Budapest, Hungary) and saved as SD files. The collected ligands were prepared by using LigPrep of Schrödinger software (Schrödinger, LLC, New York, USA; www.schrodinger.com). Tautomers and ionization states at pH 7 ± 0.2 were generated and their 3D structures minimized using the OPLS2005 force field. Only the conformer showing the lowest conformational energy was retrieved. In the case of compound **5**, two different tautomeric states were obtained: 1H-indazol (**5**) and 2H-indazol (**5t**).

Template preparation

The electron cryo-microscopy structure of rat TRPV1 at 3.4 Å resolution was used as template to model human TRPV1 by homology. The rTRPV1 was downloaded from the Protein Data Bank (PDB ID 3J5P) and prepared using the PrepWiz wizard from the Schrödinger software with the following settings: (1) removal of water molecules and artifact groups (peptide linkers); (2) addition of missing atoms and residues using the protein structure prediction program Prime [1]; (3) assignation of protonation states using PROPKA from the Schrödinger suite at pH 7.0. The processed structure was subjected to restrained energy minimization in implicit solvent using the OPLS2005 force field [2] as implemented in the Impref module of the Schrödinger. 5,000 steps of conjugate gradient/steepest descends were used and a maximum allowed root-mean-square deviation (RMSD) for heavy atoms

of 0.30 Å was applied. The minimization stopped after the energy gradient converged below 0.01 kcal/mol. The reliability of this structure was assessed in a further step using the Ramachandran plot generated with MOE v.2011.10 (MOE) [3] (Figure ESM4).

The transmembrane region of two adjacent subunits (chains A and C) used for further molecular docking simulations was obtained by removing residues L111-S402 of the intracellular region and the chains B and D from the structure.

Homology modeling

Protein sequences of rTRPV1 (UniProt ID: O35433) and hTRPV1 (UniProt ID: Q8NER1) orthologs were downloaded from the UniProt website (www.uniprot.org) and aligned using ClustlX2.1[4]. The amino acid sequence of chain A of the modeled rTRPV1 structure (template is a homotetramer) was added to this alignment (Figure ESM5). The extracellular region comprising residues N604-S626 was missing in the template and, consequently, it was not considered. The following *in silico* mutations were introduced into the template using the MOE v.2011.10 software. These mutations were: (1) I514M, V518L, V525A, S526T, Q533H, R534L, M547L, L585I into the transmembrane region of chain A; (2) L585I into the transmembrane region of chain C (Figure ESM6). In a further step, this "humanized" form of rTRPV1 was refined using the same protocol as mentioned before. The Ramachandran plot obtained for this structure showed two outliers, residues T468 and D601, both located in the extracellular loops (Figure ESM 7). However, these residues were located far from the binding site of the protein, and therefore they may not affect the performance of the docking experiments.

Docking studies

Docking studies were performed using the GOLD v.5.2.2 program (GOLD, Cambridge Crystallographic Data Center). The binding site was defined on the interface of adjacent subunits A and C as a sphere of radius 8 Å and center between the hydroxyl groups of Y511 and T550 of subunit A (Figure ESM 8). Ligands were docked into the binding site of the protein using 100 runs of the genetic algorithm and a search efficiency of 100% to ensure an exhaustive exploration of the conformational space of the ligand. No early termination was used and the diverse solutions options

were turned off. In addition, the side chains of the residues Y511, S512, L547, T550 and E570 were allowed to ±10° from the input positions, because numerous mutational studies confirm importance of these residues for binding of agonists. Generated poses were scored with the ChemPLP scoring function [5].

For each docking solution a separate protein-ligand complex in mol2 format was generated using the `gold_utils` file of the software GOLD. The resulting complexes were loaded into the software MOE using an in-house script. The energy of the protein-ligand complexes was minimized using the LigX built-in tool of the software MOE in the MMFF94x force field. Protein-ligand interaction fingerprints (PLIF)[6] were generated with the default settings in MOE (Figure ESM9).

Common scaffold clustering

The minimized docking poses were clustered according to the root-mean-square deviation (RMSD) of the heavy atoms of the common scaffold. For each class of antagonists a unique SMARTS string was used (Figure ESM10). To this aim, an RMSD matrix was calculated separately for each class of antagonists. The dissimilarity matrix was clustered with the program RStudio [7], using complete linkage as clustering algorithm and a clustering height of 2 Å. Since the fragment used for common scaffold clustering was small in comparison to the whole molecule, we kept those clusters containing at least 10 poses of any ligand docked (Figure ESM11).

Pharmacophore modeling

The pharmacophore modeling was performed in the software Ligandscout v4.1 [8]. The structure-based pharmacophores were created for a centroid pose of each common scaffold cluster with the default settings. The fit-score of the docking solutions to the current pharmacophore was calculated which represented an amount of matched pharmacophoric features by the pose. The final pharmacophore hypotheses for the binding of antagonists were derived by merging pharmacophore models of several selected common scaffold clusters.

Tables

Table ESM1 Compounds used in the docking study, their IC_{50} values and literature references.

#	ID in ChEMBLdb	IC_{50}^{Ref}, nM	#	ID in ChEMBLdb	IC_{50}^{Ref}, nM
**1**	CHEMBL436638	2[9]	**11**	CHEMBL1092853	0.21[10]
**2**	CHEMBL101751	3[11]	**12**	CHEMBL1214342	0.23[10]
**3**	CHEMBL323134	3[11]	**13**	CHEMBL457188	0.3[12]
**4**	CHEMBL419351	3[11]	**14**	CHEMBL456162	0.4[12]
**5**	CHEMBL1083396	3.5[13]	**15**	CHEMBL456778	0.5[12]
**6**	CHEMBL103898	680[11]	**16**	CHEMBL456962	380[12]
**7**	CHEMBL440535	770[14]	**17**	CID46204201*	460[10]
**8**	CHEMBL103842	1000[11]	**18**	CHEMBL1093131	665[10]
**9**	CHEMBL106391	1500[11]			
**10**	CHEMBL347492	6300[14]			

*PubChem ID

Figures

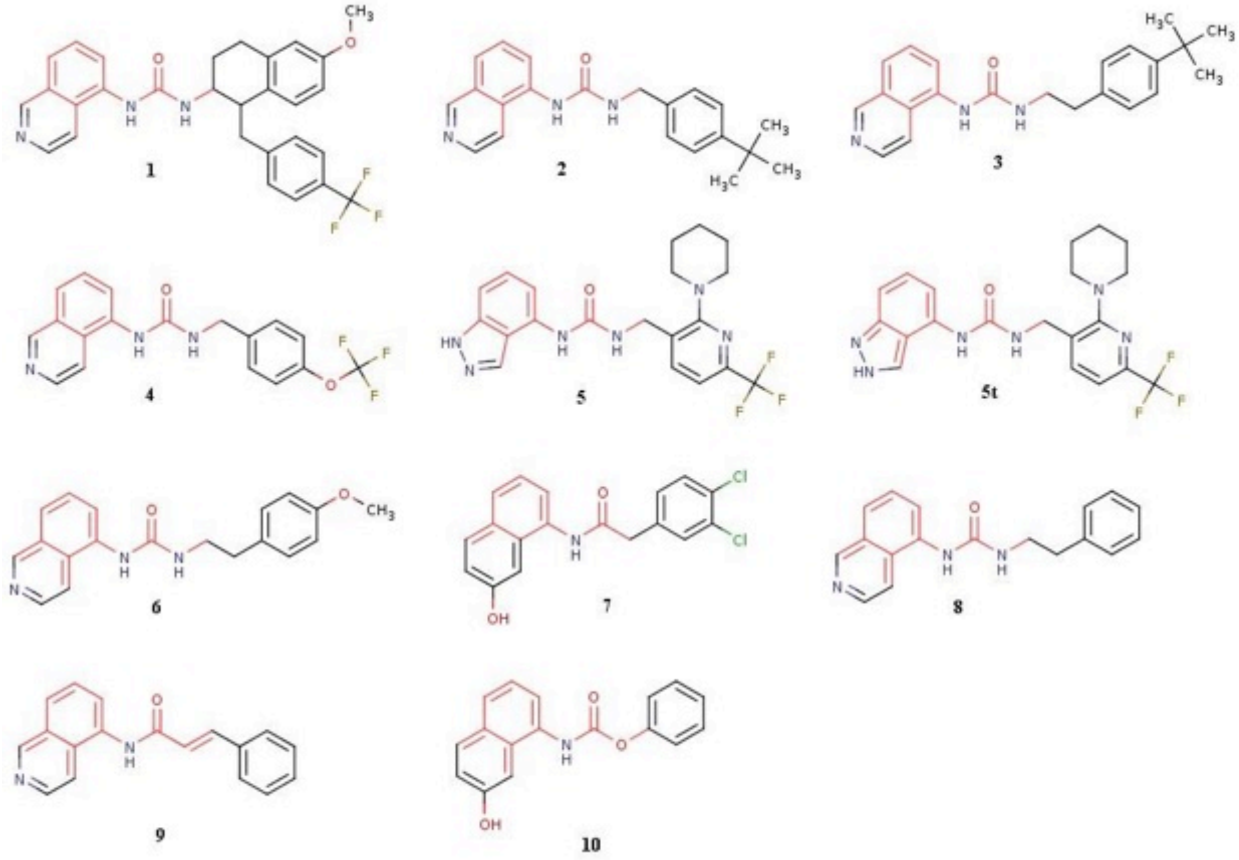

Figure **ESM2** Chemical structures of compounds of class 1; common scaffold is highlighted in red

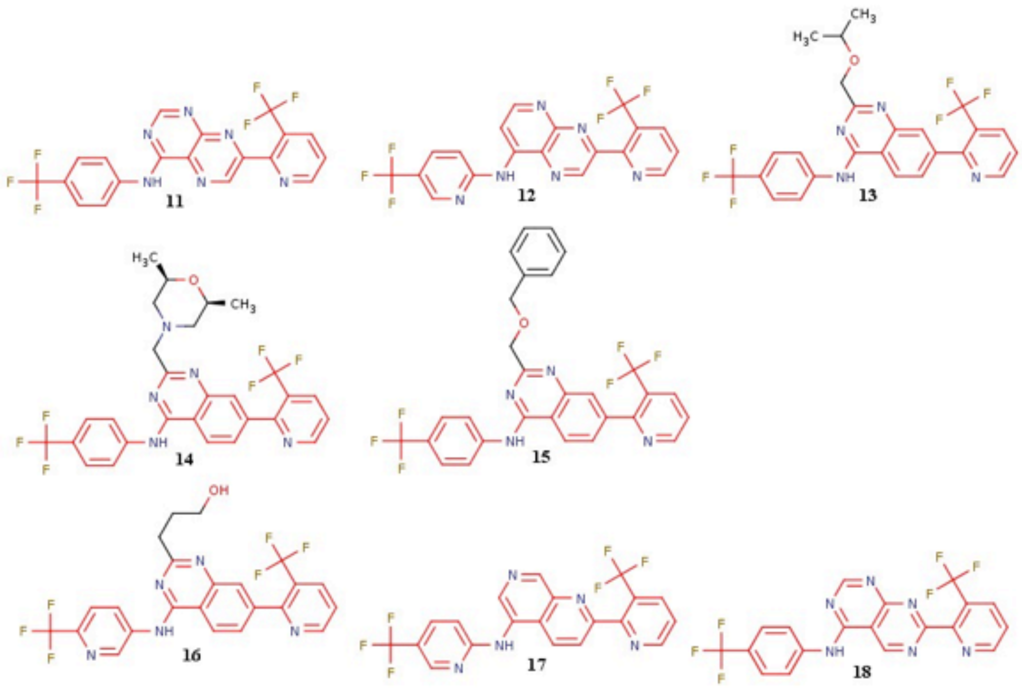

Figure **ESM3** Chemical structures of compounds of class 2; common scaffold is highlighted in red

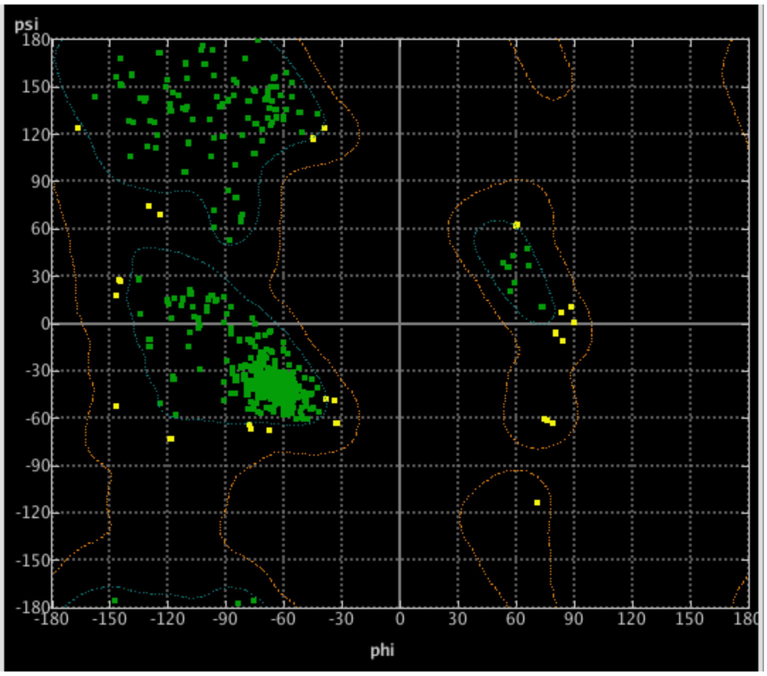

Figure ESM4 Ramachandran plot for 3J5P after energy minimization

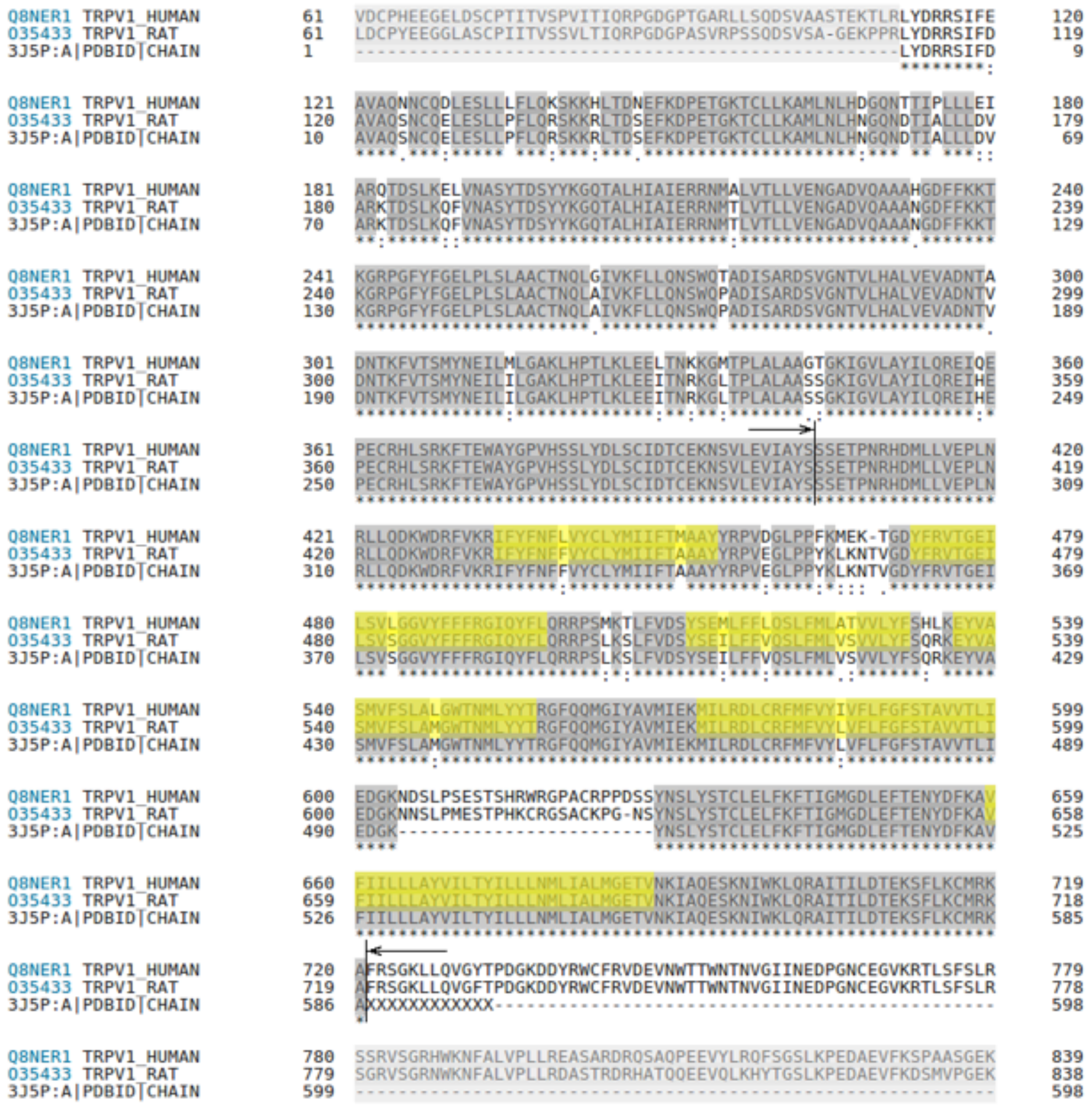

Figure **ESM5** Alignment of hTRPV1 and rTRPV1 amino acid sequences with amino acid sequence of 3J5P PDB structure; dissimilar residues are marked with white or bright yellow background; transmembrane regions are highlighted in yellow; region used for modeling is marked with black arrows

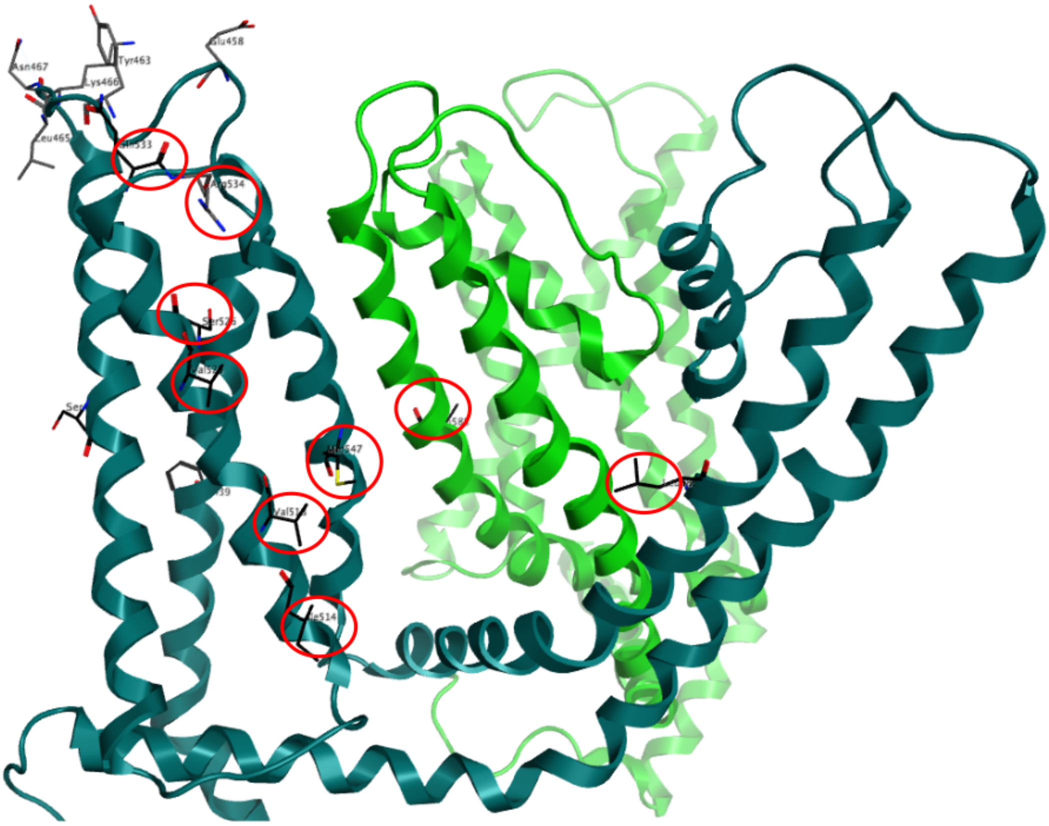

Figure **ESM6** Two adjacent subunits of rTRPV1; amino acid residues, that differ in rTRPV1 and hTRPV1 in the transmembrane region are depicted in black; residues mutated *in silico* are circled in red

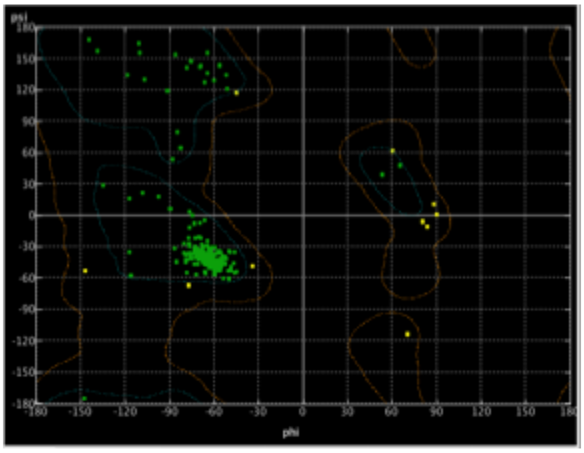

Figure **ESM7** Ramachandran plot for hTRPV1 prepared for molecular docking

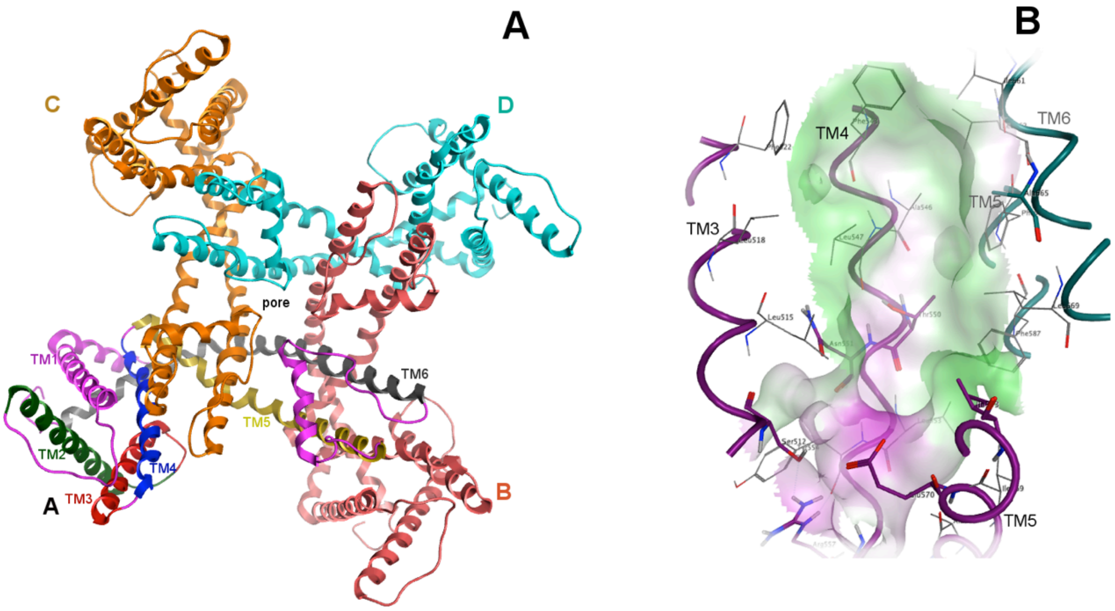

Figure **ESM8** TRPV1 structure; (**A**) View from the extracellular side; the intracellular region is removed for clarity; Four identical subunits (A, B, C and D) of TRPV1 and transmembrane regions (TM) of subunit A are marked in different colors; (**B**) Molecular surface of the agonist binding pocket is color-coded according to lipophilicity: green represents hydrophobic surface of the receptor and magenta – hydrophilic; TM regions of subunit A are in magenta and TM regions of subunit C in cyan

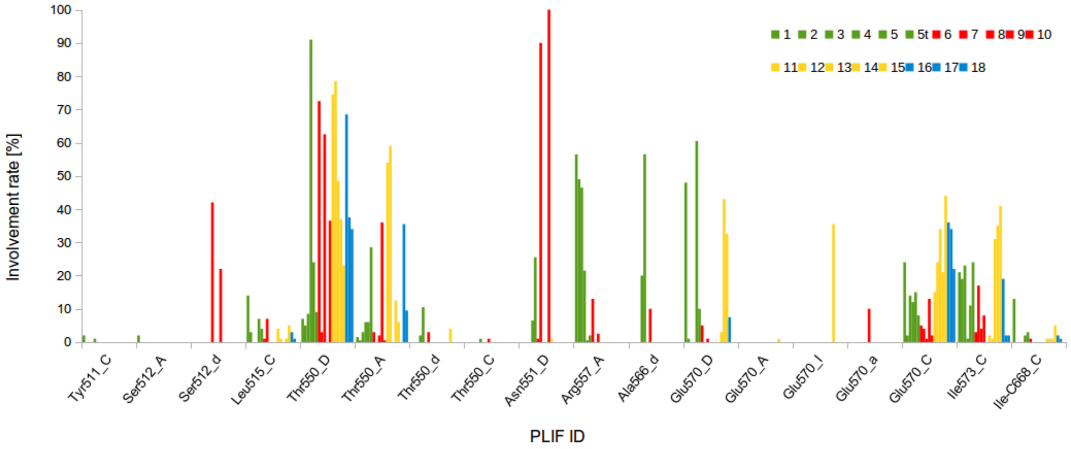

Figure **ESM9** Protein ligand interaction fingerprint (PLIF) plot obtained for the docking poses of class 1 (green: actives; red: inactives) and class 2 (yellow: actives; blue: inactives) antagonists; on the X axis, PLIF ID represents the amino acid residue and the interaction type in which it participates: A - side chain hydrogen bond acceptor, D - side chain hydrogen bond donor, a - backbone hydrogen bond acceptor, d - backbone hydrogen bond donor, I - ionic, C - surface contact (van der Waals interactions); on the Y axis, the involvement rate represents percent amount of poses participating in the interaction

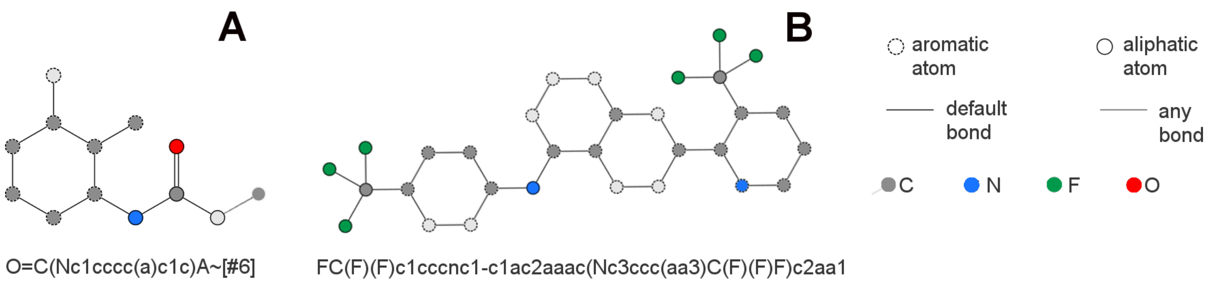

Figure **ESM10** Scaffolds and their SMARTS strings used for common scaffold clustering of antagonists of class 1 (A) and class 2 (B)

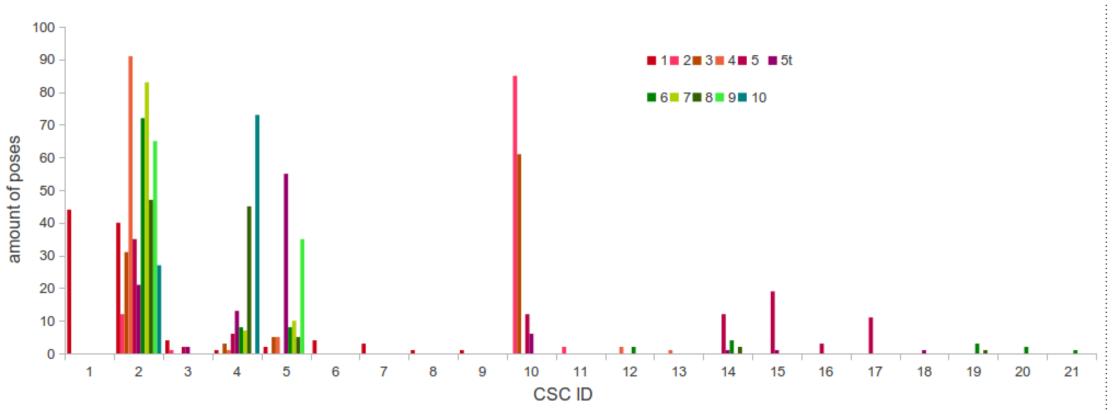

Figure **ESM11** Distribution of the docking poses of isoquinolines in CSCs; each bar is color-coded differently for each docked compound and represents the amount of its poses, which belonged to a certain CSC

References:

1. Jacobson MP, Pincus DL, Rapp CS, Day TJ, Honig B, Shaw DE, Friesner RA (2004) Proteins 55(2):351-367
2. Kaminski GA, Friesner RA, Tirado-Rives J, Jorgensen WL (2001) Journal of Physical Chemistry B 105(28):6474-6487
3. Inc. CCG. Molecular Operating Environment (MOE). 2013.08. 101 Sherbooke St. West, Suite #910, Montreal, QC, Canada, H3A 2R7; 2015
4. Larkin MA, Blackshields G, Brown NP, Chenna R, McGettigan PA, McWilliam H, Valentin F, Wallace IM, Wilm A, Lopez R, Thompson JD, Gibson TJ, Higgins DG (2007) Bioinformatics 23(21):2947-2948
5. Korb O, Stutzle T, Exner TE (2009) J Chem Inf Model 49(1):84-96
6. Labute P (2001) J Chem Comp Group
7. Racine JS (2012) Journal of Applied Econometrics 27(1):167-172
8. Wolber G, Langer T (2005) J Chem Inf Model 45(1):160-169
9. Jetter MC, Youngman MA, McNally JJ, McDonnell ME, Zhang SP, Dubin AE, Nasser N, Codd EE, Flores CM, Dax SL (2007) Bioorg Med Chem Lett 17(22):6160-6163
10. Blum CA, Caldwell T, Zheng X, Bakthavatchalam R, Capitosti S, Brielmann H, De Lombaert S, Kershaw MT, Matson D, Krause JE, Cortright D, Crandall M, Martin WJ, Murphy BA, Boyce S, Jones AB, Mason G, Rycroft W, Perrett H, Conley R, Burnaby-Davies N, Chenard BL, Hodgetts KJ (2010) J Med Chem 53(8):3330-3348
11. Jetter MC, Youngman MA, McNally JJ, Zhang SP, Dubin AE, Nasser N, Dax SL (2004) Bioorg Med Chem Lett 14(12):3053-3056
12. Blum CA, Zheng X, Brielmann H, Hodgetts KJ, Bakthavatchalam R, Chandrasekhar J, Krause JE, Cortright D, Matson D, Crandall M, Ngo CK, Fung L, Day M, Kershaw M, De Lombaert S, Chenard BL (2008) Bioorg Med Chem Lett 18(16):4573-4577
13. Brown BS, Keddy R, Perner RJ, DiDomenico S, Koenig JR, Jinkerson TK, Hannick SM, McDonald HA, Bianchi BR, Honore P, Puttfarcken PS, Moreland RB, Marsh KC, Faltynek CR, Lee CH (2010) Bioorg Med Chem Lett 20(11):3291-3294
14. McDonnell ME, Zhang SP, Nasser N, Dubin AE, Dax SL (2004) Bioorg Med Chem Lett 14(2):531-534
